# Immune precision medicine for cancer: a novel insight based on the efficiency of immune effector cells

**DOI:** 10.1186/s40880-019-0379-3

**Published:** 2019-06-14

**Authors:** Jean-François Rossi, Patrice Céballos, Zhao-Yang Lu

**Affiliations:** 1 Institut Sainte Catherine, 84918 Avignon, France; 2 Université Montpellier 1, UFR Médecine, 34396 Montpellier, France; 3 Département d’Hématologie, CHU de Montpellier, 34295 Montpellier, France; 4 Unité de Thérapie Cellulaire, CHU Saint-Eloi, 34295 Montpellier, France

**Keywords:** Precision therapy, Immunotherapy, NK lymphocytes, T-lymphocytes, Dendritic cells, Vaccination, Cancer

## Abstract

Cancer cell growth is associated with immune surveillance failure. Nowadays, restoring the desired immune response against cancer cells remains a major therapeutic strategy. Due to the recent advances in biological knowledge, efficient therapeutic tools have been developed to support the best bio-clinical approaches for immune precision therapy. One of the most important successes in immune therapy is represented by the applicational use of monoclonal antibodies, particularly the use of rituximab for B-cell lymphoproliferative disorders. More recently, other monoclonal antibodies have been developed, to inhibit immune checkpoints within the tumor microenvironment that limit immune suppression, or to enhance some immune functions with immune adjuvants through different targets such as Toll-receptor agonists. The aim is to inhibit cancer proliferation by the diminishing/elimination of cancer residual cells and clinically improving the response duration with no or few adverse effects. This effect is supported by enhancing the number, functions, and activity of the immune effector cells, including the natural killer (NK) lymphocytes, NKT-lymphocytes, γδ T-lymphocytes, cytotoxic T-lymphocytes, directly or indirectly through vaccines particularly with neoantigens, and by lowering the functions of the immune suppressive cells. Beyond these new therapeutics and their personalized usage, new considerations have to be taken into account, such as epigenetic regulation particularly from microbiota, evaluation of transversal functions, particularly cellular metabolism, and consideration to the clinical consequences at the body level. The aim of this review is to discuss some practical aspects of immune therapy, giving to clinicians the concept of immune effector cells balancing between control and tolerance. Immunological precision medicine is a combination of modern biological knowledge and clinical therapeutic decisions in a global vision of the patient.

## Introduction

The development of a disease in each individual is an inherently heterogeneous process that is determined by a unique combination of exogenous and endogenous factors. Molecular pathological epidemiology (MPE) provides a novel insight in underlying the causal mechanisms of a disease, to find an approach for individualized treatment [1–3]. According to the definition of the National Institutes of Health, precision medicine is “an emerging approach for disease treatment and prevention that takes into account individual variability in genes, environment, and lifestyle for each person” [4]. Precision medicine has become a generic term referring to techniques that evaluate either the host or the disease to enhance the likelihood of beneficial treatment outcomes from medical interventions [5]. Immune precision medicine is not only when immune therapy merges with precision medicine [6], but it also encompasses a better biological understanding of the tumor cells and its microenvironment; a better evaluation of the mechanisms implicated in immune control, immune senescence, and the different crossroads within a bio-clinical overview, in order to define a personalized therapeutic strategy [7]. Based on the concept of immune surveillance, the immune system should ideally work to eradicate cancer cells [8, 9]. However, tumors are still able to evade this system, leading to immune surveillance failure [10]. Cancer immunotherapy can be envisaged by the following four strategies to block the tumor immune evasion and to restore immune surveillance: (1) increasing the number of immune effector cells (IECs) by infusing ex vivo expanded IECs to improve the effector/tumor ratio; (2) increasing the IECs recognition affinity to tumor antigens or tumor-associated antigens (TAA); (3) improving the homing of killer IECs to the cancer cells through its microenvironment by amplifying their trafficking and homing mechanisms; (4) blocking the immune suppression ability of cancer cells. These strategies may restore the immune surveillance by not only killing the tumor cells but also preventing the emergence of new tumor cell clones which may result due to gene mutation after anti-tumor therapy.

Immune therapy was initiated in the early nineties through attenuated bacteria to create inflammatory stimuli [11]. After the Second World War, allogeneic transplantation (AlloT) was developed as a rescue strategy for radiation-induced bone marrow injury and was then introduced in the treatment for leukemias [12]. The presentation of the new immune component from the donor to a recipient made it possible to control the tumoral residual disease. The efficacy of AlloT has demonstrated in hematological malignancies, particularly for acute leukemias, and post-transplantation, where the administration of donor lymphocyte infusion (DLI) has improved the efficacy of immune therapy [13]. However, despite a modest therapeutic benefit was observed when specifically-activated and amplified immune cells were administered in certain solid tumors, AlloT failed to demonstrate major responses in solid cancers [14]; probably due to the poor accessibility of IEC to target the cancer cells. The development of immunological research has lead clinicians to directly use IEC-drugs that have been activated ex vivo to treat malignancies, and different immune adjuvants to reinforce cellular activity or inhibit specific immune checkpoints.

The aim of this review is to discuss how and when to use the different available immune therapeutic tools to support the activation, amplification, or administration of active IEC against the cancer cells.

## Pretreatment considerations: asking the right questions

A personalized and complete bio-clinical evaluation of the functional IEC is mandatory to guide the correct therapeutic choice, as to whether reinforce the IEC to kill cancer cells or to lower the number of those IEC which promotes cancer cell evasion. For making the right therapeutic choice, the following questions should be taken into consideration: (1) how to improve the patient’s care based on the evaluation of the patient’s immune status; (2) how to maintain an efficient IEC number to cancer cells ratio, (3) whether is it necessary to infuse IEC-drugs or to stimulate the patients’ IEC, (4) what is the anti-tumor activity of these IEC, their homing and presence within the cancer microenvironment; (5) how to lower the unfavorable mechanisms, particularly those that favor cancer cell growth and (6) what would be the right timing for treatment administration, and which combination or sequence of drug infusion to implement. Below, we discuss the different aspects that need to consider before giving immunotherapy.

## Evaluation of the different cancer cell aspects

### Cancer microenvironment

The interaction between tumor cells and non-tumor cells mediated by cell–cell contact and soluble molecules, such as cytokines, chemokines, growth factors, and enzymes create the cancer microenvironment [15, 16]. This is a heterogeneous medium containing both activating and blocking cells which target the tumor. We observed that IEC localization has an impact on the prognosis of patients having follicular lymphoma (FL), particularly for CD8 T-cells and T regulators (Treg or FoxP3 positive cells) [17]. In that study, the interfollicular CD8/FOXP3 positive cell ratio was significantly higher in patients with histological grade 3 tumors (2.04 vs. 1.63) and had a high-risk FL international prognostic index (FLIPI) (2.99 vs. 1.53) compared to those with grade 1–2 tumors or a low-intermediate FLIPI index. Similar results were obtained for the follicular CD8/FOXP3 positive cell ratio. The interfollicular CD8/FOXP3 ratio was found to have prognostic value (5-year overall survival [OS] of 82% vs. 59% for a ratio of ± 1.68). In addition, an interfollicular FOXP3 positive cell number of more than 86 cells/mm^2^ was correlated with a more favorable outcome (*P * = 0.03) [17]. So, modulation of these IEC could be achieved at a clinical level by administering cytotoxic T-cells or lowering Tregs by short immune suppression. The biological/chemical status of the cancer microenvironment is associated with immune response and clinical prognosis. This includes cycling hypoxia with spatial and temporal fluctuations in oxygen levels that are correlated to neo-vascularization, chemoresistance, and tumor metastasis [18, 19]. Different information from the cancer microenvironment, particularly the equilibrium state between cytotoxic/suppressive cells and their localization and functional status are necessary information for further precise immunomedical biological algorithms.

### Cancer cells antigens expression

Self-produced proteins produced in the body are non-antigenic due to self-tolerance. Proteins produced by tumors have abnormal structures and act as tumor antigens. When these are present only on tumor cells, they are known as tumor-specific antigen (TSA) and when present on both tumor cells and some normal cells, they are known as TAA. The major histocompatibility complex class II (MHCII) is responsible for presenting these tumor-derived antigen peptides to the T-lymphocytes [20]. Targeting these cancer antigens is the key for anti-tumor immune therapy and for increasing efficacies, identifying the density of these antigenic peptides is important.

### Evaluation of tumor mass and its reduction possibility

Tumor mass evaluation became a major parameter for patient follow-up in the context of clinical research as well as in standard medicine. A great number of tools have been developed to perform such analysis, from standard medical imaging to the evaluation of minimal residual disease by flow cytometry or next-generation sequencing. Dynamic evaluation of the tumor mass is a major parameter to define the right choice of the immune tools and the right time to use them. This is based on the balance of efficiency between the number of cancer cells and the number and efficacy of killer IECs [21]. Immune therapy was generally used after tumor mass debulking to control residual disease. Nowadays, due to the efficacy of the new immune tools including IEC, the therapeutic choice needs to be guided by this new concept, the balance of efficiency between tumor mass and the number of IEC, needing dynamic evaluation.

### Cancer immune-evasion mechanisms

The mechanisms by which cancer cells evade the immune system should be thoroughly considered, and to include the evaluation of the functional status of the cancer cells, their proliferative and metastatic mechanisms, their metabolic status, the MHC expression which is responsible for displaying the tumor-derived antigen peptides on the antigen-presenting cells (APCs), and the mutanome that transforms mutated tumoral proteins into neoantigens, leading to immunome [22–24]. The aim is to reinforce the immune system to destroy the cancer cells by blocking their protective mechanisms. The Food and Drug Administration (FDA) has approved the anti-programmed death-1 (PD-1) monoclonal antibody (mAb), pembrolizumab, for any solid tumor overexpressing the PD-1 receptor ligand, which is associated to microsatellite instability-high, a condition of genetic hypermutability which generates mutated proteins which are not recognized as self-proteins and represent neoantigens [25]. It was the first time that a tissue-agnostic drug was approved by the FDA based on tumor genetics and may represent conditions to combine vaccination and immune checkpoint inhibitors.

## Evaluation of the patient immune system

The first step of precision immune medicine is to evaluate the functions of the patients’ immune system, with the aim of determining its ability to respond to therapeutic interventions. These include the assessments of the genetic context of the patient, particularly the autoimmune processes that may require gene polymorphism analysis, the evaluation of immunosenescence based on the immune functions such as tryptophan metabolism and cytokine production, and immune exhaustion including the evaluation of the potential consequences of chemotherapeutic or targeted drugs on the patient’s condition. Further, the existing inflammatory processes within the patient may have a major impact on immune responses, immune resistance, and metastatic process through activation of neovascularization and myeloid-derived suppressor cells in the cancer microenvironment [26]. They may have developed upon the interactions between cancer cells and their microenvironment, microbiota or due to existing co-morbidities [27, 28]. The C-reactive protein (CRP) can be used to evaluate the inflammation condition [29] and may provide hints on the immune status of the patients.

The metabolic status of the immune cells also changes under diverse conditions and is associated with modified functions of the T-lymphocytes [30]. MHC-I modulation due to the changes in tumor cell metabolism regulates the tumor sensitivity to cytotoxic T-lymphocytes (CTL) and to natural killer lymphocytes (NK) through extracellular-signal-regulated kinase 5 (ERK5) expression that modulates MHC-I complex expression and influences the T- and NK activities [31]. Standard evaluation by flow cytometry of the circulating IEC is currently used but is poorly correlated to the immune status within the cancer microenvironment. The evaluation of cellular specific response against TSA after vaccine therapy could be performed by demonstrating a restricted immune T-cell response including T-cell receptor (TcR) rearrangement, ELIspot or other techniques [32].

## Choosing IEC targeting

The products to interfere in anti-tumor effect supported by the immune system can be classified into cell-containing products and non-cell-containing products. Non-cell-containing products include monoclonal antibodies (mAbs), cytokines and cytokine inhibitors, some chemical immune modulators, and anti-tumor vaccines pulsed with different adjuvants [33–38].

Immune effector cells, particularly αβT-, γδT-, NK-, and NKT-lymphocytes, are immune cells that mainly support cancer immune surveillance. In cancer patients, autologous IECs are generally inactive because the activity and/or the number of these cells are reduced by tumor cells in the tumor microenvironment. One therapeutic possibility is to improve the activity or the number of IEC to bypass the mechanisms of immune failure, by using immune modifiers or by switching specific antigen presentation. Likewise, Allo-innate cells, including NK lymphocytes could be used because of their non-MHC recognition mechanism and stress protein expression, such as the heat shock proteins (HSP), in tumor cells.

In the early eighties, clinicians used cytokines, such as interleukin (IL) 2 [39], to expand autologous immune cells, particularly T-lymphocytes [40–42]. Autologous lymphocyte-activated killer cells (LAK) and cytokine-induced killer (CIK) lymphocytes, derived from the peripheral blood of the patients can be expanded ex vivo, and then re-infused into them [43]. In this way, the tissues surrounding the cancer cells were more likely to contain a larger number of immune cells, particularly tumor-infiltrating lymphocytes (TIL) with anti-cancer activity. TILs taken from tumor tissue obtained after biopsy were then considered as antigen-specific T-lymphocytes, and were cultured and expanded in the lab before their re-infusion [44]. However, the major problem of such a product was the uncontrolled cell heterogeneity including both specific cytotoxic cells and Tregs that may favor tumor progression. This cellular heterogeneity may explain the lack of major clinical benefit. Specific anti-cancer eradication is supported by the recognition of TAA presented by APCs that triggers specific T-lymphocytes [45–48]. Epigenetic modifying agents such as deacetylase inhibitors may modulate TAA expression and the release of mutated proteins representing neoantigens which may promote vaccination therapy [49, 50]. Recent advances in active cellular therapies have shown that by genetically modifying the T-lymphocytes, their efficacy through the antigen receptor (TCR) or tumor cell linkage via chimeric antigen receptor (CAR) could be enhanced [51]. As such, modern technology is paving the path to amplify the immune system, which can become better equipped with more effective IEC against cancer cells. Below, we review and discuss the different IEC.

### αβ T-lymphocytes

Cytotoxic T-lymphocytes expressing αβ TcR represent the main subset of circulating lymphocytes in which the MHC-restricted antigen recognition is mandatory for their activity. LAK, TIL and some CIK lymphocytes mentioned above are mainly αβ T lymphocytes. Autologous CTL, being predominantly αβ T-lymphocytes, for instance, the CAR-T lymphocytes and TcR-engineered T-lymphocytes, have been genetically engineered to specifically target and to destroy cancer cells.

CAR-T lymphocytes were developed by generating a genetic construct that encodes the antigen-binding region of a mAb and the intracellular components of the TcR that activate signals upon binding to target cell surface antigens. Different generations of CAR-T lymphocytes were developed based on the combination of the antigen-binding domains of the heavy and light chains of antibodies that are fused to the CD3-ζ intracellular signaling domain and co-stimulatory molecules to enhance the avidity of T-lymphocytes for antigens [51]. Using the CAR-T lymphocytes as a therapeutic strategy has been associated with clinical success in hematological malignancies including acute myeloid leukemias by using chimeric CD123 [the IL3-Rα chain] receptor-modified T-lymphocytes [52], chronic lymphocytic leukemia (CLL) [53], pre-B acute lymphoblastic leukemia with CD19, and other lymphoid malignancies [54]. However, in B-acute lymphocytic leukemia, the median event-free survival was 6.1 months, and 26% of patients developed severe cytokine release syndrome [55] with fatal outcome observed [56]. Recently, kinetics and biomarkers have been identified, with the description of a classification-tree algorithm to guide studies and to propose therapy including anti-IL-6 [57, 58]. There have been more than 70 clinical trials with CAR-T lymphocytes referred at the National Institute of Health (NIH) [59]. The response rate superior to 80% observed in refractory lymphoid malignancies has led several pharmaceutical companies in Western countries and China to invest in the CAR-T strategies [60]. In contrast, the TcR engineering is based on the ability to genetically modify lymphocytes to express TcR with specificity against a chosen antigen that allows these chimeric TcR-T cells to target tumor cells with MHC class I-restricted antigen peptide presentation. This methodology was first used with MART-1 antigen in melanoma but demonstrated a lower response rate (13%) as compared to that observed with TIL (51–72%) [61].

### γδ T-lymphocytes

γδT-lymphocytes represent a minor subset (10%) of circulating lymphocytes, with a dominant Vγ9Vδ2 TcR cell subpopulation, that recognizes the endogenous pool of isopentenyl pyrophosphate. γδT-lymphocytes are implicated in anti-viral and anti-tumor responses, and in modulating immune response [62–64]. Like NK lymphocytes, γδT-lymphocytes respond to stimulation by stress- and/or infection-induced ligands. Usually, these ligands are weakly- or not-expressed, and are up-regulated in the presence of stress or infection, leading to cytotoxicity by binding to the NK Gene 2D (NKG2D) receptor or by direct recognition [65]. In addition, γδT-lymphocytes also express pattern recognition receptors (PPR), such as Toll-like receptors (TLR) that modulate their activation. γδT-lymphocytes exert their activity through a TcR-independent recognition of non-peptidic phosphorylated antigens, called phosphoantigens (PA) produced by many bacteria such as different strains of *Staphylococcus*, *Enterococcus faecalis*, *Myxococcus fulvus*, *Streptococcus mutans*, *Lactobacillus casei*, and *Lactobacillus plantarum*, or derived from the mevalonate isoprenoid pathway [66]. γδT-lymphocytes express CD16 (FCγRIII) receptor, leading to antibody-dependent cell-mediated cytotoxicity (ADCC) [67]. In hematological malignancies, large inter-individual variations in the expansion capacity of γδT-lymphocytes have been observed among different patients having multiple myeloma (MM), non-Hodgkin lymphoma (NHL), and CLL [68]. Our group has demonstrated that γδT-lymphocytes could be amplified by both biphosphonates and IL2, and in vitro observations have shown that these allogeneic cells could kill both human MM cell lines and fresh myeloma tumoral cells from patients [69]. The anti-tumor effect of γδT-lymphocytes has been mainly demonstrated in lymphoid malignancies [70, 71]. Two strategies have been applied, including adoptive cell transfer after in vitro expansion, particularly used in solid cancers, and in vivo therapeutic activation of γδT-lymphocytes by PA or aminobiphosphonates with low dose IL-2 that has been preferentially used in hematological malignancies. Using both therapeutic strategies, the response rate was variable due to the limited number of patients included in these studies but the clinical benefit was observed when combined with other treatments, for instance, AlloT [72].

**Fig. 1:**
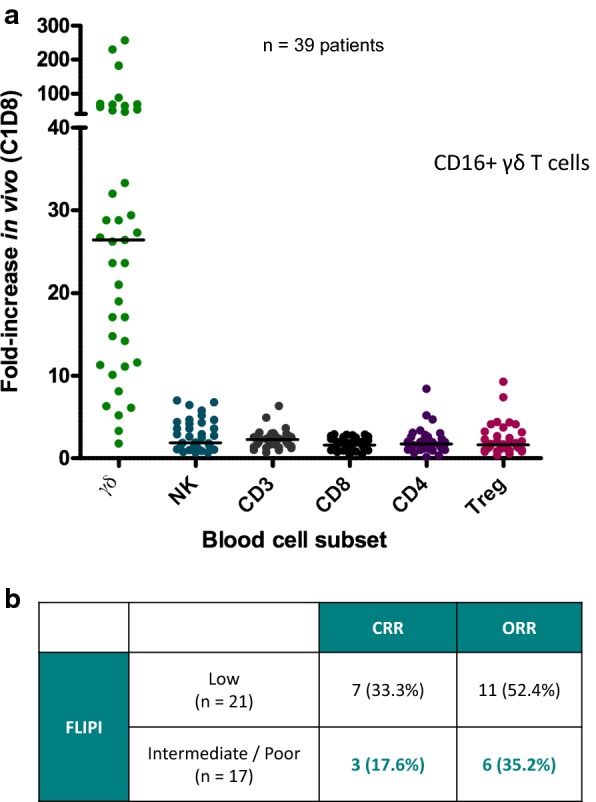
Results of the phase II study combining in vivo γδ T-cells stimulator (IPH1101), interleukin 2 and rituximab for patients having follicular lymphoma. The patients were treated with bromohydrin pyrophosphate (BrHPP, Innate Pharma Inc. Marseille, France, at 750 mL/m^2^, 3 times every 3 weeks), low dose IL-2 (8 MIU, daily for 5 days, every 3 weeks) and rituximab (375 mg/m^2^, 4 times weekly). There was a dramatic increase of circulating T-lymphocytes without any amplification of other lymphocyte subpopulations including Tregs. **a** The change of circulating immune cell subpopulations at day 21 as compared to day 0. **b** Complete response rate (CRR) and objective response rate (ORR) in patients having FL depending on the FLIPI (Follicular Lymphoma International Prognostic Index), i.e., low index and intermediate/poor index. IL2: interleukin 2; Treg: T-regulator; NK: natural killer lymphocytes; C1D8: cycle 1 Day 8

To demonstrate that γδT-lymphocytes are also amplified in vivo, we have conducted a multicenter phase II clinical study comprising of 45 patients who presented with advanced FL in relapse or refractory relapse, since last line of therapy, with a median time of 19.1 months [Protocol No. IPH1 101-202, EudraCT No. 2006-006891-39, presented at EHA 2010;95:p10]. The patients received bromohydrin pyrophosphate (BrHPP, named IPH1101 from Innate Pharma Inc. Marseille, France), at 750 mg/m^2^ 3 times every 3 weeks, low dose IL2 (8 MIU, daily for 5 days, every 3 weeks) and rituximab (375 mg/m^2^, 4 times weekly) by intravenous route. The median age of the patients was 59 years (range 39–74 years), and 47% of them had a low FLIPI and 27% had high FLIPI. Selective amplification of targeted CD16^+^ γδT-lymphocytes was observed, peaking after the first cycle of therapy (Fig. 1a). No amplification of the Treg lymphocytes was observed, but they demonstrated a tendency to increase slightly after the third cycle of the treatment. The objective response rate (ORR) was 47.4%, with the highest ORR observed in patients with low FLIPI (Fig. 1b). The tolerance was satisfactory, with 90% of the adverse events being grade 1–2, possibly due to the use of IL2. No biomarker was correlated to clinical response, except a concentration of rituximab superior to 25, 000 ng/mL at day-86 as previously reported [73, 74]. In vitro synergism of γδT-lymphocytes was also observed with a new generation of anti-CD20 mAbs like obinutuzumab [75]. We observed that γδT-lymphocytes were able to amplify the activity of other immune cells, particularly the NK lymphocytes and dendritic cells (DC) [76, 77]. These results showed the efficacy and non-toxic effects of γδT-lymphocytes to shrink tumor mass, possibly due to their non-MHC-restriction with no graft versus host (GVH) effect, thereby leading to the opportunity for new clinical trials regarding the CAR γδT-lymphocytes [78].

### NK lymphocytes

Natural killer lymphocytes are defined as CD3^−^ CD56^+^ lymphocytes, with different circulating subpopulations, particularly CD56^bright^ and CD56^dim^ lymphocyte subsets. NK lymphocytes can rapidly kill target cells independently to prior immunization or MHC restriction, rending these cells attractive for immune therapy [79]. This activation process is regulated by a balanced mechanism between the inhibitory and activating signals from different receptors, including NK cytotoxicity receptors (NCR), C-type lectin receptors (NKG2A/B, NKG2C, NKG2D, NKG2F) and killer immunoglobulin-like receptors (KIR) [79], leading to greater responsiveness to subsequent activation stimuli through a process called NK cell licensing [80, 81], with the possible generation of long-lived memory cells [82]. More than 740 clinical trials, corresponding to different origins of the NK, from peripheral blood, umbilical cord blood, progenitors or NK-92 cell line [83], with different modes of amplification/activation of autologous or Allo- (including haplo-identical) NK, are referred at the NIH. The first clinical trial with NK lymphocytes was based on autologous amplified NK lymphocytes by using in vivo cytokines, i.e., IL2, IL12, IL15, IL18, IL21 and type I interferon (IFN) [84]. By using such stimulations, cells became LAK, or CIK if γIFN was added to the culture conditions 24 h before stimulation by anti-CD3 with IL2 [85, 86]. CIKs are represented by a mixture of T-cells, NK T-cells and NK lymphocytes which particularly express CD16, CD45RA, CR7, CD27, CD28. The number of cytotoxic cells needed for lysing tumor cells is 2 × 10^11^ for LAK and only 0.5–5 × 10^10^ for CIKs [87]. Cytotoxic T-cells are effective against cancer cells, but also responsible for graft-versus-host disease (GVHD) effect. In Allo cellular products, the minimal contamination of T-cells to avoid GVHD has been defined from Allo bone marrow transplantation at lower than 1 × 10^3^/kg [88].

Autologous NK lymphocytes have been used in both solid and hematological malignancies with minor clinical efficacy but demonstrated better efficacy with haplo-identical-Allo NK lymphocytes [89, 90]. As such, haplo-identical or AlloNK lymphocytes (usually with KIR-mismatch) were mainly administered with AlloT lymphocytes in hematological malignancies, or in combination with mAbs (i.e., rituximab, trastuzumab, antiGD2 F8, elotuzumab). Different amplification/activation procedures were used, with cytokines (IL2, IL12, IL15, IL15, and IL21) with or without K562-m15-41BB ligand engineered cell line as feeder cells, generally using CD56-selection and/or CD3/CD19 depletion and followed by in vivo cytokine administration (IL2 or IL15) or IL2DT fusion protein to deplete Treg that express high amount of IL2R α chain [90]. To activate autologous NK in vivo, other therapeutic procedures have been developed by using an anti-KIR mAb with no clinical efficacy when used alone, or by inhibiting disintegrin and metalloproteinase domain-containing protein 17 (ADAM17) to improve CD16 expression [91–93]. In most of the clinical trials, NK cell-drugs were prepared from a donor to a recipient. Umbilical cord blood offers the possibility to obtain NK cellular products from progenitor CD34^+^ cells as shown by Glycostem Inc. (Hertogenbosch, Netherland), or by amplifying and activating mixture of naïve NK lymphocytes from the umbilical cord blood [94]. CAR-NK have been developed in pre- and early clinical studies, with redirected human NK and NK-92 cell line, against CD19, CD20, CD38, CD138, CD244, human epithelial growth receptor (HER) 2, disialoganglioside (GD) 2, epithelial cell adhesion molecule (EPCAM), CCND3 Subset (CS)1, lectin, mannose-binding (LMAN)1 [95, 96].

Due to their capacity for killing cancer cells, the use of activated NK and CAR-NK lymphocytes represent an attractive anti-cancer cell-drug for both hematological malignancies and solid tumors.

#### NK T lymphocytes

NKT cells are a subpopulation of lymphocytes possessing the phenotypic properties of both T- and NK-lymphocytes, which makes them attractive for immune therapy. Invariant NKT, also named as type I NKT, lymphocytes expressed specific TcR that recognizes lipid antigens presented by the conserved and non-polymorphic MHC class I like molecule CD1d and receptors for cytokines such as IL2, IL8, IL23 and IL25 [97]. Several drugs have been generated for triggering these cells through CD1d molecules, including α-galactosylceramides synthetic analogs, but no major clinical effect was observed [98]. To direct cancer cell lysis, invariant NKT lymphocytes was used to activate both innate and adaptive immune cells in the tumor microenvironment, through the secretion of cytokines, such as Th1-, Th2- and Th17-type responses, particularly the γ-chain cytokine or CD132 (i.e., IL2, IL4, IL7, IL9, IL15 and IL21-R). In addition, the adoptive transfer of type I NKT lymphocytes generated in vitro from autologous lymphocytes or from Allo-CD34 progenitors cells isolated from umbilical cord blood using IL15 and stem cell factor in preclinical models have demonstrated promising results [99].

## Activation of IEC and restoring anti-tumor immune functions

### Activation of IEC’s anti-tumor effect by DCs

**Fig. 2:**
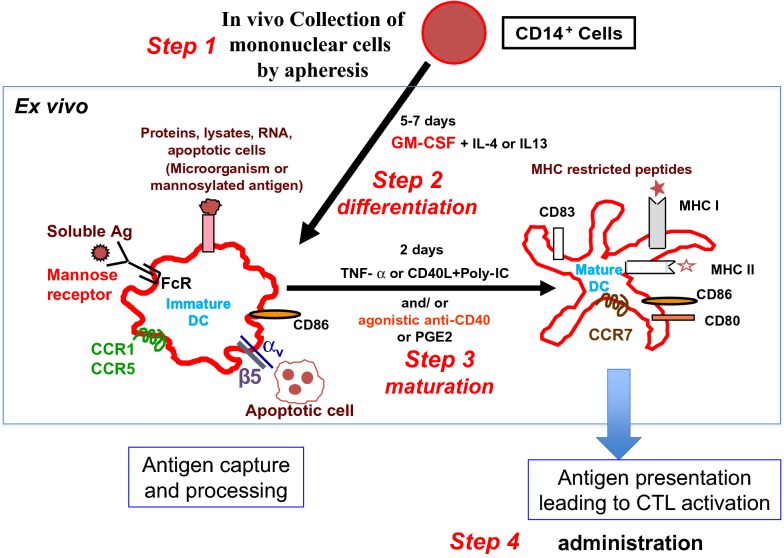
Ex vivo maturation and differentiation of dendritic cells (DC) from monocytes. Mononuclear cells were collected by apheresis from the patient. These mononuclear cells were differentiated ex vivo to immature DC in the presence of GM-CSF and IL4 or IL13. The mature DCs were obtained after stimulation by TNF-α and they exhibited different biomarkers, such as CD86, CD80, CCR7, and the antigen-MHC complex. GM-CSF: Granulocyte–Macrophage Colony Stimulating Factor; IL: interleukin; TNF: Tumor Necrosis Factor; MHC: major histocompatibility complex; CTL: cytotoxic lymphocytes; CCR: C–C chemokine receptor; Ag: antigen; α_v_: integrin alpha V; β5: integrin beta 5; CTL: cytotoxic lymphocytes; DC: dendritic cells; FcR: Fc receptor; Poly-IC: polycytidylic acid; RNA: ribonucleic acid

**Fig. 3:**
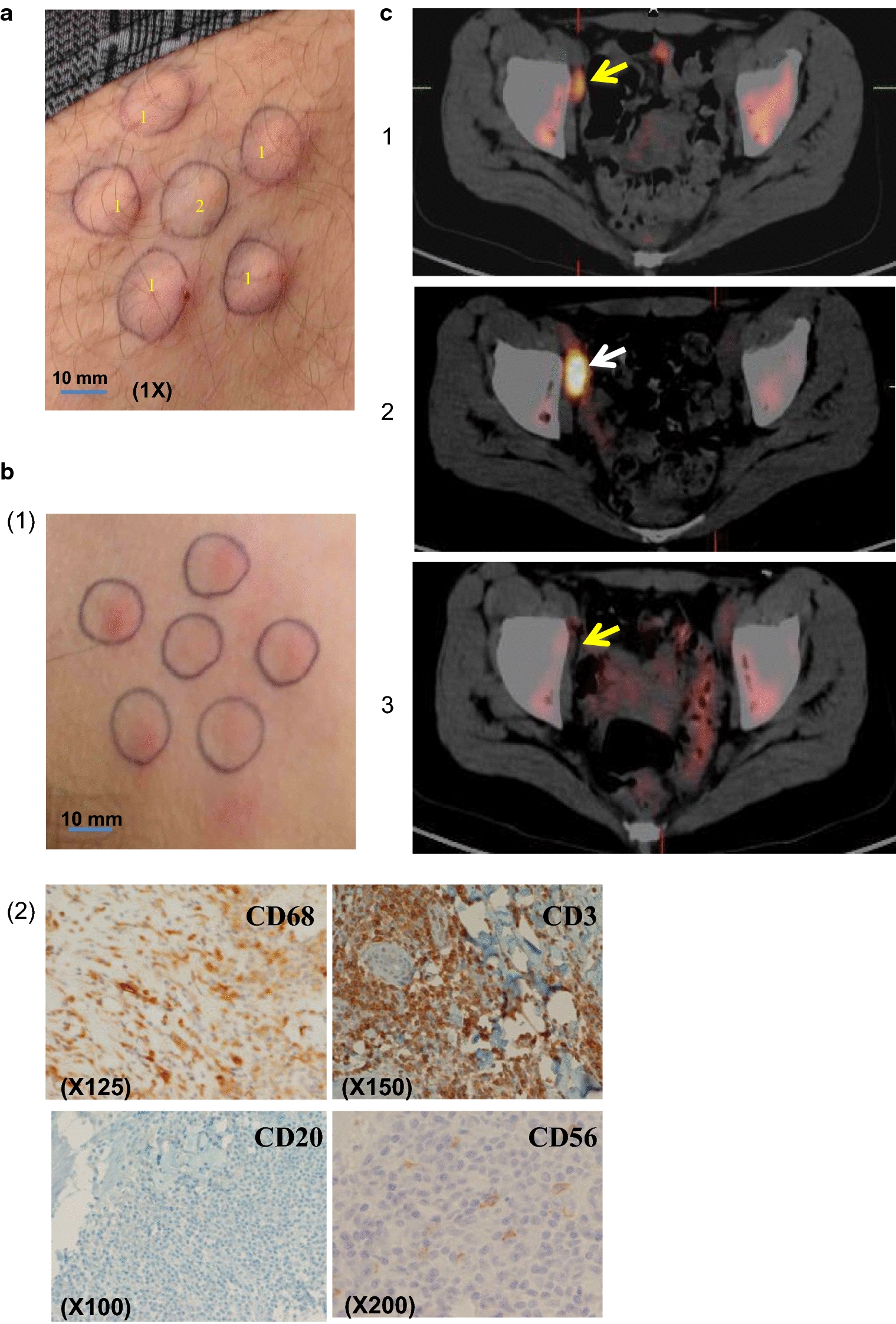
A phase II clinical trial with DC pulsed with tumoral cell lysates from patients having follicular lymphoma. **a** Intradermal injection of 0.2 mL of DC-pulsed with tumoral lysates (5 × 10^6^ DC/mL) (5 sites) and KLH intradermal injection (100 µg in 0.2 mL) used as a control. Magnification: ×1; **b** Delayed hypersensitivity after intradermal injections (1) and cutaneous biopsies (2) showing important CD3 positive infiltrate and CD68 positive macrophages; **c** [18F] fluorodeoxyglucose-positron emission tomography scanning (FDG pet-scan): (1) before vaccination with tumoral lymph node (yellow arrow), (2) 15 days after the vaccination with increased hypermetabolism of the lymph node (white arrow), (3) 2 months after the vaccination with the disappearance of the hypermetabolism (yellow arrow), suggesting an activation of the IECs within the lymph node. 1: injection site with 0.2 mL of DC-pulsed with tumoral lysates; 2: injection site of control; DC: dendritic cells; KLH: Keyhole limpet hemocyanin

Mature autologous DCs are professional APC, generated from mononuclear cells (MC) by using a cocktail of cytokines [100]. DC stimulated specific CD8 positive cells against TSA. Autologous DC pulsed with TAA or cell lysates have been obtained ex vivo, with the expression of different surface markers and the secretion of diverse cytokines and chemokines, as previously described [101] and illustrated in Fig. 2. In metastatic prostate cancer, activated and pulsed DCs have been generated ex vivo from autologous MC, by using a prostatic acid phosphatase-granulocyte macrophage colony stimulating factor (GM-CSF) fusion protein, the Sipuleucel-T. This cellular product was used in a randomized study, demonstrating a 4.1-month in median survival difference observed in favor of the vaccination arm as compared to the control arm [102]. Vaccination programs using TSA alone or DC-pulsed with TSA have been extensively used in different cancers, particularly using idiotype shared by cancer cells in FL, but with little clinical benefits [103–105]. As the multitargeting approach is considered to be more active against cancer cells, we used DC-based therapy pulsed with tumor cell lysates in a phase II study for patients in relapse having FL [106]. A specific response was demonstrated after intra-dermal injections (Fig. 3a, b). Eleven patients were treated, of whom two had complete responses (CR) before vaccination and 2 had CR after the vaccination program. An early (15 days after vaccination) [18F] fluorodeoxyglucose-positron emission tomography was performed in one patient, and it showed an early metabolic activation in the lymph node (Fig. 3c), that may constitute an early clinical indicator of the efficacy among the immunomonitoring.

Clinical responses regarding the use of DCs have been disappointing, with an ORR of around 15% [107]. Improvements may include the use of immune adjuvants that improve the antigen delivery as mineral salts, emulsions, and liposomes, or immunostimulants such as TLR-ligands (particularly TLR7/8 agonists such as the imidazoquinoline family [108], cytokines, saponins, bacterial exotoxins and exosomes [109–112]. Recently, boosting the capture and presentation of TAA were developed by using hydroxylapatite combined to tumor extract containing HSP from a frozen sample after thawing, then subcutaneously injected, disposal named APAVAC^®^ (URODELIA Inc, Toulouse France) [113]. A randomized placebo-controlled double-blinded chemo-immunotherapy clinical trial was conducted in a pet dog model of diffuse large b-cell lymphoma using APAVAC^®^. The median time to progression and median lymphoma-specific survival were significantly different in dogs having chemo-immunotherapy vs. chemotherapy alone; 304 days vs. 41 days (*P *=0.0004) and 505 days vs. 159 days (*P *= 0.0018), respectively [114]. Preliminary data in humans have been published with promising data in advanced cancers as shown by Ciocca et al. [113].

The efficiency of DC migration from the skin to the lymph nodes has been studied and is linked to the maturation status and the C–C chemokine receptor (CCR) 7 expression of the DCs [115]. Recently, a pilot clinical trial was reported with a personalized vaccine generated by autologous DCs pulsed with whole-tumor cell lysate in patients having ovarian cancer [116]. This vaccine elicited efficiently a broad antitumor immunity, including private neo-antigens. Tumor cells eradication has been achieved by using in situ vaccination with a TLR ligand and anti-OX-40 antibody in an animal model [116].

**Fig. 4:**
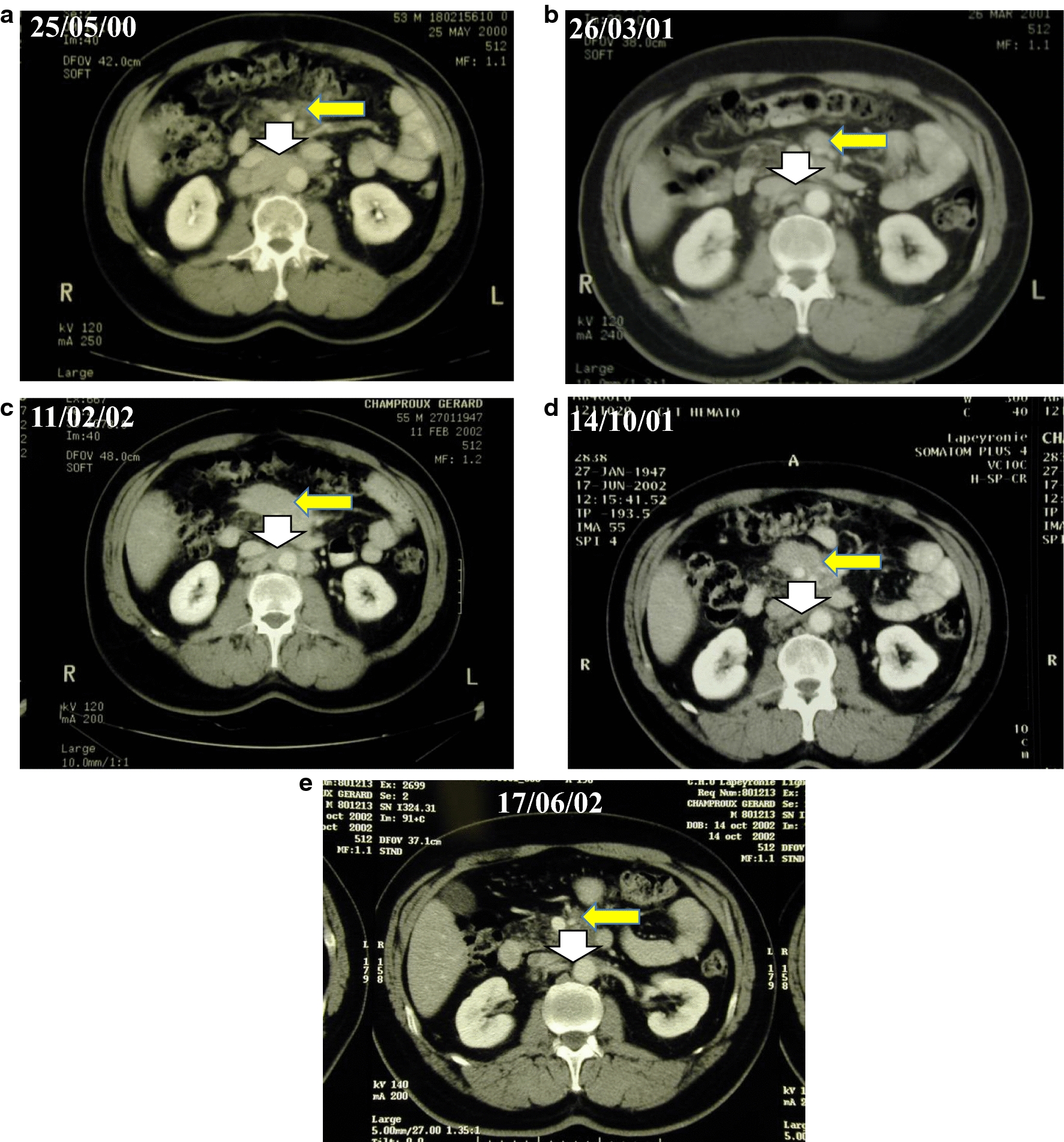
Delayed complete response observed in a patient receiving GM-CSF and rituximab, suggesting an in vivo vaccination. A 58 years-old Male was diagnosed in December 1996 as follicular lymphoma grade II, stage IIIAb, FLIPI 2 with high tumor burden. He received mini-CHOP and interferon alfa followed by a partial response. His disease progressed in March 1998 and he was treated by three courses of DHAP and high dose therapy with autologous transplantation associated with a complete response. He relapsed in May 1999 and received rituximab with partial response. After reprogression in May 2000 (**a**), he received GM-CSF and rituximab with a long-lasting regression and fluctuation of the tumor over 2 years, as observed on the following scanners (**b**–**d**) and no tumor on **e**, suggesting an in vivo vaccination. He reprogressed in October 2006 and was treated by rituximab with stabilization for 1 year. FLIPI: Follicular Lymphoma International Prognostic Index; CHOP: cyclophosphamide, doxorubicin, vincristine, prednisone; DHAP: dexamethasone, cytarabine, *cis*-platinum; GM-CSF: Granulocyte–Macrophage Colony Stimulating Factor

Important interactions between IECs has led to an amplified or synergistic activity among the different immune therapies supporting combined therapies. The aim of such combined strategies is to amplify specific recognition to reinforce the killing activity or to lower immune suppressive effect. TAA or MHC class I expression at the cell surface of cancer cells could be reinforced by epigenetic modulation or by interfering on metabolism pathways [23, 30, 31, 117]. Cell crosstalk is essential for immune therapy. Subsets of CD56^bright^CD16^dim/−^ NK lymphocytes present in lymph nodes possess helper role in the production of γIFN that may improve the DC activity [118]. By combining mAbs and certain cytokines that activate DCs, we observed a delayed but progressive clinical response which may be explained by an in vivo vaccination process, as suggested in one patient treated by rituximab and GM-CSF [119] (Fig. 4). This observation suggested that rituximab possibly enhances the apoptosis of tumor cells, and GM-CSF participates to in vivo generation of mature DC and may prolong disease immune control.

### Blocking the immune suppression source of cancer cells

**Fig. 5:**
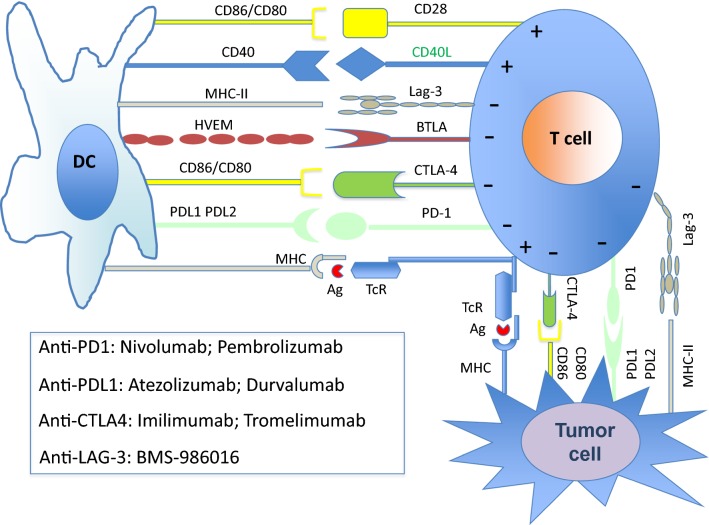
Illustration of the immune checkpoint molecules and their inhibitors. Immune checkpoint inhibitors are potent molecules implicated in the cell–cell communications. They are present on T-cells and their ligands are shared by tumor cells and dendritic cells (DC). Immune checkpoint molecules send negative signals to T cells and inhibit the later activities. Monoclonal antibodies have been developed against different molecules and used as therapeutic checkpoint inhibitors. CTLA-4: cytotoxic T-lymphocyte antigen-4, TcR: T cell receptor; MHC: major histocompatibility complex; HVEM: Herpes Virus Entry Mediator; PD1: programmed death; PDL-1/2: programmed death ligand-1/2; Lag-3: lymphocyte-activation gene 3; BTLA: B- and T-lymphocyte attenuator; Ag: antigen

The T-cells can be activated by two signals in normal physiology. One when the T-cell receptor is bounded to an antigen-MHC or second, when the T-cell surface receptor, CD28, binds to CD80/CD86 proteins [120]. CTLA4 inhibits the binding of CD80/CD86 to CD28 and negatively regulate the activation of T-cells. The immune response can be enhanced by blocking immune checkpoints shared by T-cells with the presence of their ligands on both cancer cells and DCs (Fig. 5) [121, 122]. The expression of immune checkpoints by TIL, and particularly Treg, results in low responsiveness of the immune control of cancer cells. Thereby, targeting these markers are very attractive targets for reversing immune tolerance, particularly in cancer. The anti-CTLA-4 mAb, ipilimumab, received the FDA approval in March 2011 for the treatment of metastatic melanoma [123], metastatic renal cell carcinoma, and non-small cell lung carcinoma [124], as it has shown efficacy in causing a shift increase in the number of cytotoxic T-cells to enhance anti-tumor response. Further development is ongoing in both solid tumors and hematological malignancies with different molecules (MDX-1106 or nivolumab, and CT-011 or pidilizumab as anti-PD-1; MDX-1105 as anti-PD-L1, and other molecules directed against T-cell Immunoglobulin and mucin domain-containing protein 3 [Tim-3], lymphocyte activation gene-3 [LAG-3], and V-domain Ig-containing suppressor of T-cell activation [VISTA]). More than 92 clinical trials regarding immune checkpoint inhibitors are now referred at the NIH [125].

## Defining the therapeutic strategy

The therapeutic strategy must be clearly defined based upon proper analysis regarding the characteristics of the cancer cells, the tumor-host microenvironment, and the immune system status of the patient. Different therapeutic options are available aiming to stimulate IEC activity, to block immune checkpoints or immune cells that favor cancer cell growth. Lymphodepletion is generally used to modify the host microenvironment, particularly to limit the Treg activity [126]. This can be achieved by giving total body irradiation, non-myeloablative chemotherapy as conditioning regimen for AlloT or prescribing a low dose of chemotherapy such as cyclophosphamide, 5-fluorouracil, fludarabine, gemcitabine, bortezomib, sorafenib and sunitinib [127].

Other targeted therapies have been described to have an immune effect. Recently, idelalisib, an inhibitor of phosphoinositide 3 kinase δ [128] has been mentioned to be able to deplete Treg and myeloid-derived suppressor cells [129], and ibrutinib, to favor antigen presentation [130]. Other therapeutic strategies have been developed, including molecules targeting Treg, such as anti-CD25 molecules, anti-Foxp3 or anti-CTLA4, inhibitors of STAT3 that regulates the expression of TGF-β and IL10 cytokines or inhibitors of indoleamine 2,3-dioxygenase [127, 131].

**Fig. 6:**
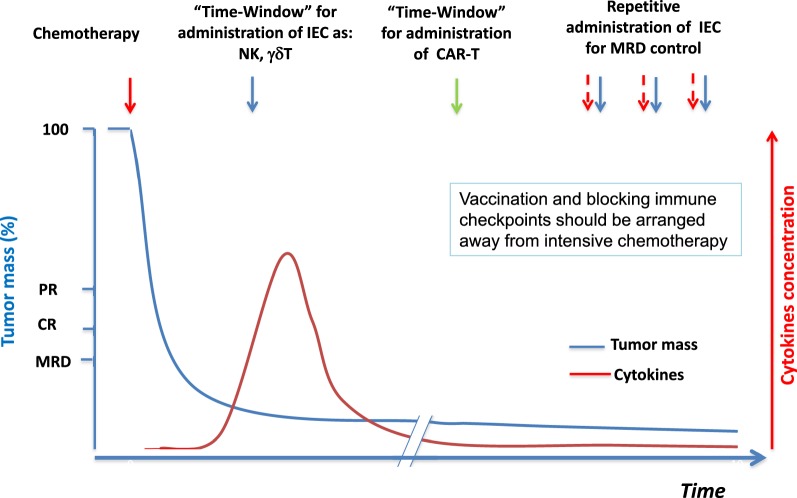
Time-windows of opportunity in immune therapy for cancers. After tumor mass reduction by chemotherapy, there is a burst of cytokines and lymphodepletion that are benefitting for in vivo activity of the immune effector cells such as NK or γδ T-lymphocytes. CAR-T cells are engineered cytotoxic T-cells that could be used instead of chemotherapy when chemoresistance is observed or to complete tumor cell killing. To control residual disease, repetitive administration of immune effector cells could be proposed. Immune checkpoint inhibitors are potent immune adjuvants to be combined for amplifying the T-cell response, probably at a lower dose and to avoid immune exhaustion. The role of vaccine using neoantigens is probably an alternative therapy for frail patients and this represents a good opportunity to challenge the immune specific functions along with the immune control of the tumor. IEC: immune effector cells; CAR-T: chimeric antigen receptor T-cell therapy; MRD: minimal residual disease; PR: partial response, CR: complete response

Strategy for Immune therapy requires choosing the best immune therapeutic tools at the right time, as suggested in Fig. 6. In addition, a combination of immune and standard treatments is also crucial, meaning that radiotherapy or chemotherapy may favor the circulation of neoantigens generated after cancer cell killing, to be collected for vaccine programs. After high dose therapy in patients having MM, our group has described a burst of cytokines, particularly of IL7/IL15 at day-8 and IL6 at day-15 [132]. Autologous transplantation offers another “window” of opportunity for using IECs. High dose therapy followed by autologous transplantation is generally associated with the induction of a pre-apoptotic status for both the tumor cells and the CD3 positive cells that could be saved by cytokine peaks. This opens two therapeutic opportunities. One, to block IL6 which is the survival/proliferation factor of MM cells, as described in one of our previous study [133], and/or second, to administer activated/amplified IEC after high dose therapy, including γδT, NK, or CAR-T lymphocytes [134]. Similarly, AlloT and particularly haplo-identical AlloT are considered as a model for immune therapy.

## Conclusion

Immune precision medicine is a complex medical trail, starting from a careful personalized evaluation of the characteristics of the cancer cell and the conditions of the cancer-host microenvironment as well as the bio-clinical status of the host. Personalized evaluation of the disease opens the way to identify the optimal therapeutic strategy. The aim is to reinforce the IECs that kill cancer cells and to decrease those factors that favor cancer cell growth. These can be achieved, in a dynamic way, by administering IECs such as CAR-lymphocytes, to kill tumor cells. Then, the immune system could be challenged to eradicate residual disease with vaccination whose activity can be amplified by inhibitors of immune checkpoints and/or immune adjuvants. Based on such, we firmly believe that immune precision medicine is internal medicine with modern tools that are beneficial for improving the treatment and outcomes of cancer patients.

## Data Availability

Not applicable.

## Abbreviations

ADAM-17: disintegrin and metalloproteinase domain-containing protein 17
ADCC: antibody-dependent-cell cytotoxicity
AlloT: allogeneic transplantation
APC: antigen presenting cell
BrHPP: bromohydrin pyrophosphate
CAR: chimeric antigen receptor
CCR7: C–C chemokine receptor 7
CD: cluster of differentiation
CIK: cytokine induced killer
CLL: chronic lymphocytic leukemia
CR: complete response
CRP: C reactive protein
CS1: CCND3 subset 1
CTL: cytotoxic T-lymphocytes
CTLA-4: cytotoxic T-lymphocyte antigen-4
DC: dendritic cell
DLI: donor lymphocyte infusion
EPCAM: epithelial adhesion molecule
ERK 5: extracellular-signal-regulated kinase 5
FDA: Food and Drug Administration
FLIPI: Follicular Lymphoma Prognostic Index
GD2: disialoganglioside 2
GMCSF: granulocyte macrophage colony stimulating factor
GVHD: graft versus host disease
HER2: human epithelial growth receptor 2
HSP: heat shock protein
IEC: immune effector cells
IFN: interferon
IL: interleukin
IR: inhibitory receptors
IV: intravenous
LAK: lymphocyte activated killer
LMAN1: lectin mannose binding 1
mAb: monoclonal antibody
MHC: major histocompatibility complex
MPE: molecular pathological epidemiology
MM: multiple myeloma
NHL: non-Hodgkin’s lymphoma
NIH: National Institute of Health
NK: natural killer
ORR: overall response rate
PA: phosphoantigens
PD: programmed cell death
PDL: PD ligand
PPR: pattern recognition receptors
TAA: tumor associated antigen
TcR: T-cell receptor
TGF: transforming growth factor
TIL: tumor infiltrating lymphocyte
TLR: Toll-like receptor
TNF: Tumor Necrosis Factor
Treg: T-regulator
